# Comparative efficacy and safety of holmium laser enucleation of the prostate (HoLEP) using moses technology and standard HoLEP: A systematic review, meta-analysis, and meta-regression

**DOI:** 10.1016/j.amsu.2022.104280

**Published:** 2022-08-12

**Authors:** Muhammad Zaniar Ramadhani, Yudhistira Pradnyan Kloping, Ilham Akbar Rahman, Niwanda Yogiswara, Johan Renaldo, Soetojo Wirjopranoto

**Affiliations:** a Department of Urology, Faculty of Medicine, Universitas Airlangga, Indonesia; b Dr. Soetomo General-Academic Hospital, Mayjen Prof. Dr. Moestopo No.6-8, Surabaya, 60286, Indonesia

**Keywords:** Prostatic hyperplasia, Moses HoLEP, Holmium laser enucleation of the prostate, HoLEP

## Abstract

**Purpose:**

The use of HoLEP was associated with steep learning curve thus prolonging operative procedure. The problem of learning curve could be solved with the invention of Moses HoLEP. This study aimed to evaluate the comparison of efficacy and safety between Moses HoLEP and standard HoLEP in BPH patient.

**Materials and methods:**

Systematic search was carried out using PRISMA guideline. Pubmed, Scopus and Embase were searched to collect randomized controlled trials and observational studies. Quantitative analysis was performed to evaluate the comparison in intraoperative, postoperative and complications characteristics. RevMan 5.4 and STATA were used in data analysis.

**Results:**

Total of 7 studies (1226 patients) were included. Regarding intraoperative characteristics, Moses HoLEP provided significantly shorter enucleation time (MD: 3.00, 95% CI: 5.57 to −0.43, p = 0.02), shorter hemostasis time (MD: 3.79, 95% CI: 5.23 to −2.34, p < 0.00001), and shorter laser use time (MD: 2.79, 95% CI: 5.03 to −0.55, p = 0.01). For postoperative characteristics, Moses HoLEP possessed significantly lower PVR (MD -34.57, 95% CI -56.85 to −12.30, p = 0.002). Overall complication was higher in standard HoLEP although the result was not significant (MD 0.68, 95%CI: 0.38 to 1.21, p = 0.19). Moses HoLEP possessed more superiority over standard HoLEP regarding shorter hemostasis time with the increasing of prostate size (coefficient −0.894, p = 0.044).

**Conclusion:**

Moses HoLEP demonstrated shorter enucleation time, shorter hemostasis time and shorter laser use time. Moses HoLEP also possessed lower PVR. There were no safety issues in Moses HoLEP compared with standard HoLEP.

## Highlights

• Moses HoLEP is able to divide the laser into 2 waves enabling to increase the efficiency of the HoLEP procedure.
• Moses HoLEP demonstrated superiority intraoperatively with shorter enucleation time, hemostasis time and laser use time.
• Moses HoLEP demonstrated superiority in post operative outcome of lower PVR.
• Moses HoLEP possessed no safety issues compared to standard HoLEP.

## 1 Introduction

Benign prostate hyperplasia (BPH) is a disease caused by the proliferation of the benign prostate gland, which mostly affects older men, as many as 50% of men aged 60 years. Approximately 1 in 5 men with BPH accounts for significant clinical symptoms within 1 year since the first initiation of treatment. This disease also represents as the seventh highest 1-year disease-specific medical cost. Considering this together, the burden from health care of BPH is not trivial [1]. The incremental medical costs from this condition were observed in the recent years [2]. The etiology of BPH is still not fully known, however it is suggested to be influenced by age, family history, hormonal conditions, increased inflammation, and metabolic syndrome. An enlarged prostate causes urinary problems or commonly known as Lower Urinary Tract Symptoms (LUTS), including decreased urine output, nocturia, and urgency, which possesses the potential to reduce the patient's quality of life. Treatment options for BPH ranged from watchful waiting, medical therapy such as alpha blocker, minimally invasive procedures, and open surgery [3,4].

Transurethral Resection of the Prostate (TURP) is gold standard operative treatment in mild to moderate enlargement of the prostate (30–80 ml). However, in large prostates, TURP has a higher rate of morbidity, complications, and repeated procedures. This is due to the presence of transurethral resection (TUR) syndrome if the duration of the TURP procedure prolonged. Moreover, many complications caused by TURP procedure include sexual dysfunction, retrograde ejaculation, recurrent urinary retention, and urethral stricture [4,5].

Since its introduction in 1998, holmium laser enucleation of the prostate (HoLEP) has become one of the minimally invasive therapeutic modalities which can be utilized for the treatment of BPH. The enucleation technique allows HoLEP to be used on all prostate sizes with good safety, efficacy, and durability. Therefore, in the current guidelines, HoLEP is the recommended standard therapy for the management of BPH in large prostates. However, currently the use of HoLEP is still considered suboptimal. This is due to steep learning curve prolonging the procedure, causing higher intraoperative bleeding, and increased complications [5,6].

Along with technological advancements, especially in the development of holmium laser technology, the steep learning curve which is the main obstacle in the use of HoLEP may be overcome. One of the laser technology developments that could be utilized is the Moses laser technology. Moses developed by Lumenis is able to divide the laser wave into 2 waves. The first energy wave separates the water by forming a bubble cavity, and the second wave was to transfer the laser energy through the bubble cavity directly to the target. These mechanisms were believed to increase the efficiency of the HoLEP procedure [6,7].

The evidence of Moses HoLEP in the treatment of BPH is still few. For this reason, a systematic review, meta-analysis and meta-regression were conducted to compare the use of Moses technology in the HoLEP (m-HoLEP) procedure with standard HoLEP with respect to intraoperative, postoperative and complication outcome.

## 2 Material and methods

### 2.1 Search strategy

**Fig. 1:**
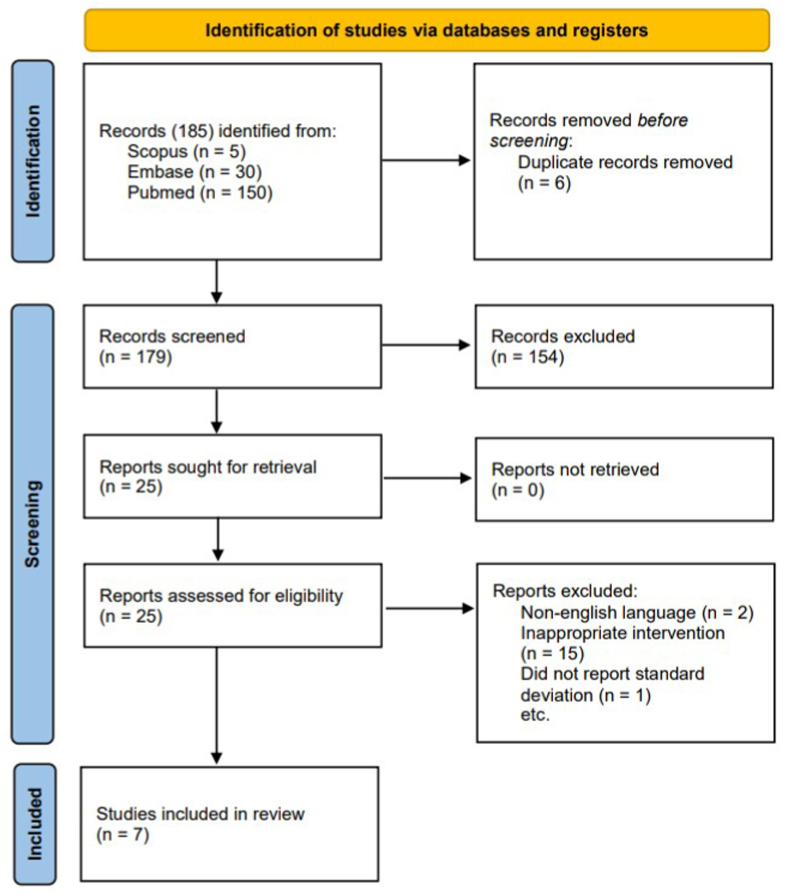
PRISMA flow diagram in the systematic search.

Up to January 2022, several databases comprised of Pubmed, Embase and Scopus were searched for clinical study (randomized controlled trial or observational study) evaluating the efficacy and safety of Moses HoLEP and standard HoLEP. The following keywords were used by combining several terms including “Moses HoLEP” OR “Moses holmium laser enucleation of the prostate” AND “holmium laser enucleation of the prostate” OR “standard HoLEP” OR “standard holmium laser enucleation of the prostate” AND “Benign prostatic hyperplasia”. Additional database was also involved to search for additional studies. There were no limitation of language, country, or publication year in this study. The search strategy was shown in Fig. 1. The protocol of this meta-analysis was registered in PROSPERO (CRD42021266151) and research registry (reviewregistry1403). This study also followed the guideline of PRISMA 2020 [8].

### 2.2 Inclusion and exclusion criteria

Studies of randomized or non-randomized controlled trials which is in accordance with the following criteria were included in this systematic review and meta-analysis: The studies were included in this meta-analysis if they met following criteria: (1) the study evaluated a comparison between Moses HoLEP and standard HoLEP, (2) the study provided intended outcome consisting of intraoperative, postoperative and complications, (3) full-text study, and (4) English language article. The studies in the form of abstract, review article, and case report were excluded.

### 2.3 Quality assessment

Two authors independently evaluated all identified inclusion studies, and any disagreement between authors was resolved through the involvement of a third reviewer. Cochrane Risk of Bias (RoB) Tools 2 was used to assess RCT study. Newcastle Ottawa Scale (NOS) was used in the assessment of retrospective/observational study. The quality of this systematic review was also evaluated and assessed using AMSTAR 2 criteria [9].

### 2.4 Data extraction

Several baseline data were extracted from the inclusion studies including author, study design, sample size, publication year, population, Prostate-specific antigen (PSA) value, prostate weight, prostate volume, Body Mass Index (BMI), age, and intervention. The primary outcome in this study was the intraoperative characteristic consisted of total operative time, enucleation time, hemostasis time, laser use time. The secondary outcome was postoperative characteristic and complications rate. Postoperative characteristics comprised of post-void residual volume (PVR), peak urinary flow rate (Qmax) and The International Prostate Symptom Score (IPSS).

### 2.5 Statistical analysis

Heterogeneity between studies was assessed using I^2^ test and p value. Heterogeneity was considered high if P value ≤ 0.05 and an I 2 value ≥ 50% and random-effects model was performed. Heterogeneity was considered low if P value ≥ 0.05 and I^2^ ≤ 50 and fixed-effects model was performed. Continuous data were extracted in the form of mean and standard deviation and were pooled into mean difference. Dichotomous data were extracted and were pooled into Odds Ratio. Meta-regression was performed to assess the relationship between intraoperative time and prostate size. Egger regression test and Begg rank correlation test were undertaken to assess the risk of publication bias. For random effects model, sensitivity analysis was conducted to assess the consistency of outcome when low-quality and highly heterogeneous trials were included in the analysis. The analysis of this study was performed using RevMan 5.4 (Cochrane Collaboration, UK) and STATA ®16 (StataCorp LLC, US).

## 3 Results

### 3.1 Search results and study characteristics

PRISMA 2020 flowchart guide was implemented as a guide in the systematic search from several databases including Embase, Scopus, and Pubmed. A total of 185 articles were generated from the pre-defined keywords. After duplicates articles were removed and screening through titles and abstracts was performed based on the pre-defined PICO, a total of 178 articles were excluded. Full-text screening was carried out to evaluate the inclusion and exclusion criteria. Finally, 7 studies comprising of 2 RCTs and 5 observational retrospective studies were included in the present study. All included study evaluated the comparison of the use of Moses HoLEP (m-HoLEP) and standard HoLEP in the treatment of BPH. All included studies in this meta-analysis were published between 2020 and 2021. All patients comprised with large BPH with the size of more than 80 cc. The total patients in this study were 914 patients. The detailed study characteristics were shown in Table 1.

**Table 1:** Baseline characteristics of included studies.

Study	Study design	Population	PSA value		Prostate weight (gram)				Prostate size (ml)		BMI	Age (years)	Total sample	Intervention
			m-HoLEP	s-HoLEP	m-HoLEP			s-HoLEP	m-HoLEP	s-HoLEP				
Kavoussi NL (2021)	*Double blind* RCT	BPH patient undergoing Moses and Standard HoLEP	6.1 ± 2.6	5.6 ± 2.5	131 ± 41			153 ± 58	>80		29.25	69.35	60 patients (Moses 30, Standard HoLEP 30)	Lumenis 120 H dual pedal laser unit Moses HOLEP, 550 μm fibers, energy 2J and frequency 20–40 Hz
Nevo A (2020)	*Double blind* RCT	BPH patient undergoing Moses and Standard HoLEP	NR		58				107		NR	68 (55–78)	27 patients (Right lobe 27, Left lobe 27)	Lumenis pulse 120H, Moses 2.0 HOLEP 550 μm end fibre laser
Large T (2020)	Retrospective	BPH patient undergoing Moses and Standard HoLEP	8.39 ± 5.9	5.86 ± 4.8	76.7 ± 77.1			72.5 ± 49.6	155.6 ± 50.3	110.5 ± 85.5	27.85	71.1	100 patients (Moses 50, Standard HoLEP 50)	Lumenis Pulse 120H, Moses HOLEP 550 μm fibers, energy 2 J dan frequency 40 Hz
Nottingham CU (2021)	Retrospective	BPH patient undergoing Moses and Standard HoLEP	NR		77		73		NR		27.9	71.5	104 patients (Moses 54, Standard HoLEP 50)	Lumenis Pulse 120H, Moses 2.0 Holep 550 μm fibers, energy 2 J dan 40 Hz
Klett DE (2021)	Retrospective	BPH patient undergoing Moses and Standard HoLEP	NR		67		61		98 (69–124)	89 (65–120)	28	71.4	435 patients (Moses 255, Standard HoLEP 180)	Lumenis Pulse 120H, Moses 550 μm fibers, energy 2J and frequency 40 Hz
Mark A. Assmus (2021)	Retrospective	BPH patient undergoing Moses and Standard HoLEP	NR		NR				124.5 (51.8–161.3)	107.5 (79.8–129.6)	28.3	72.3	188 patients (Moses 93, Standard HoLEP 95)	Lumenis 120H, Moses 2.0, energy 2J and frequency 40 Hz
Matthew S. Lee (2021)	Retrospective	BPH patient undergoing Moses and Standard HoLEP	NR		114.8 ± 73.2	115.8 ± 90.4			NR		28.67	70.5	312 patients (Moses 192, Standard HoLEP 120)	Lumenis 120H, Moses 2.0, 550 μm laser fiber, energy 2J and frequency 40 Hz

NR: not reported.

### 3.2 Risk of bias analysis

*Cochrane ROB Tools for Randomized Trials* instrument was used to evaluate the risk of bias from the RCT and the NOS to evaluate retrospective comparative studies. The *Cochrane* ROB *Tools* 2 was used to evaluate 2 RCT studies [10,11]. Kavoussi et al. was assessed as low risk of bias based on the five domains. However, the evaluation of Nevo et al. resulted as some concerns due to the D1 domain did not explain the process of allocation concealment from the study. Newcastle Ottawa Scale instrument evaluated 5 retrospective studies [[12], [13], [14], [15], [16]]. The evaluation of NOS instrument was classified into 3 groups which are low quality (0–3), medium quality (4–6) and high quality (7–9) [17]. In this study, the risk assessment of bias resulted in a score of at least 6 which indicated that the included studies had a quality assessment of the risk of bias with a minimum of moderate. The detailed assessment of risk of bias was shown in Supplementary materials.

### 3.3 Intraoperative results

**Fig. 2:**
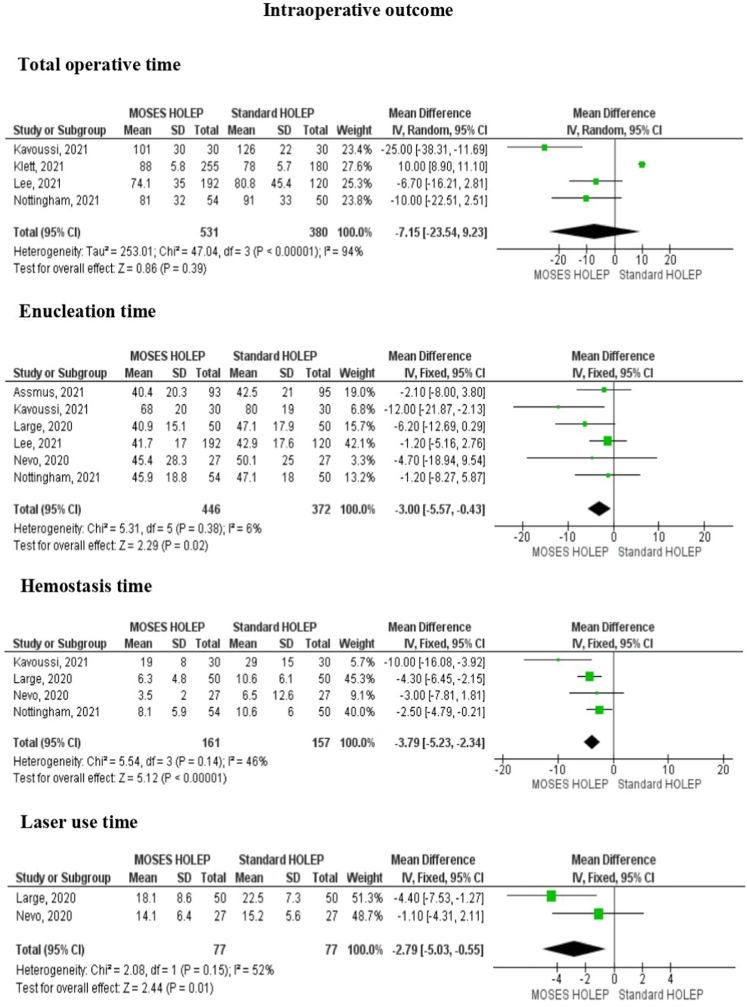
Forest plot of intraoperative outcome consisted of total operative time, enucleation time, hemostasis time, laser use time.

There were 4 outcomes in the assessment of intraoperative results. These were total operative time, enucleation time, hemostasis time and laser use time. The result of the forest plot revealed that in comparison between Moses HoLEP and standard HoLEP, there was no significant difference in total operative time (MD: 7.15, 95% CI: 23.54 to 9.23, p 0.39). However, Moses HoLEP was significantly associated with shorter enucleation time (MD: 3.00, 95% CI: 5.57 to −0.43, p = 0.02), shorter hemostasis time (MD: 3.79, 95% CI: 5.23 to −2.34, p < 0.00001), and shorter laser use time (MD: 2.79, 95% CI: 5.03 to −0.55, p = 0.01). Fig. 2 showed the detailed forest plot of intraoperative characteristics.

### 3.4 Post-operative results

**Fig. 3:**
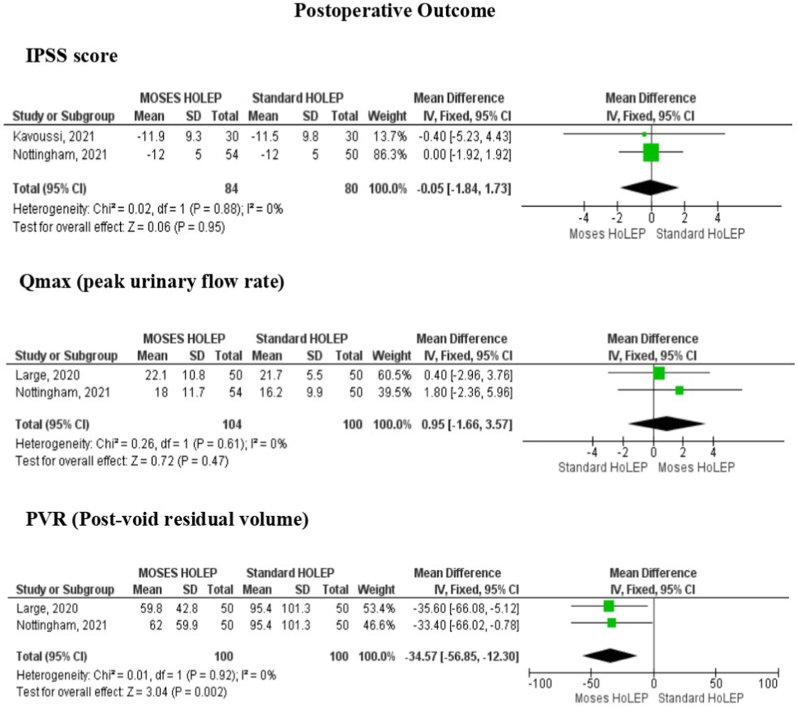
Forest plot of postoperative outcome consisted of IPSS score, Qmax (peak urinary flow rate), Post-Void Residual Volume (PVR).

*Change in IPSS*. Fig. 3a demonstrated that no significant difference was revealed between the 2 groups regarding change in IPSS between Moses HoLEP and standard HoLEP (MD: 0.05, 95% CI: 1.84 to 1.73, p = 0.95).

*Qmax*. There was no significant difference evaluated between Moses HoLEP and standard HoLEP in Qmax after the operative procedure (MD: 0.95, 95% CI: 1.66 to 3.57, p = 0.47) as shown in Fig. 3b.

*PVR*. The statistically significant difference suggested a benefit of Moses HoLEP over standard HoLEP in post-void residual volume in which Moses HoLEP possessed significantly less PVR compared to standard HoLEP (MD -34.57, 95% CI -56.85 to −12.30, p = 0.002) as shown in Fig. 3c.

### 3.5 Complications

**Fig. 4:**
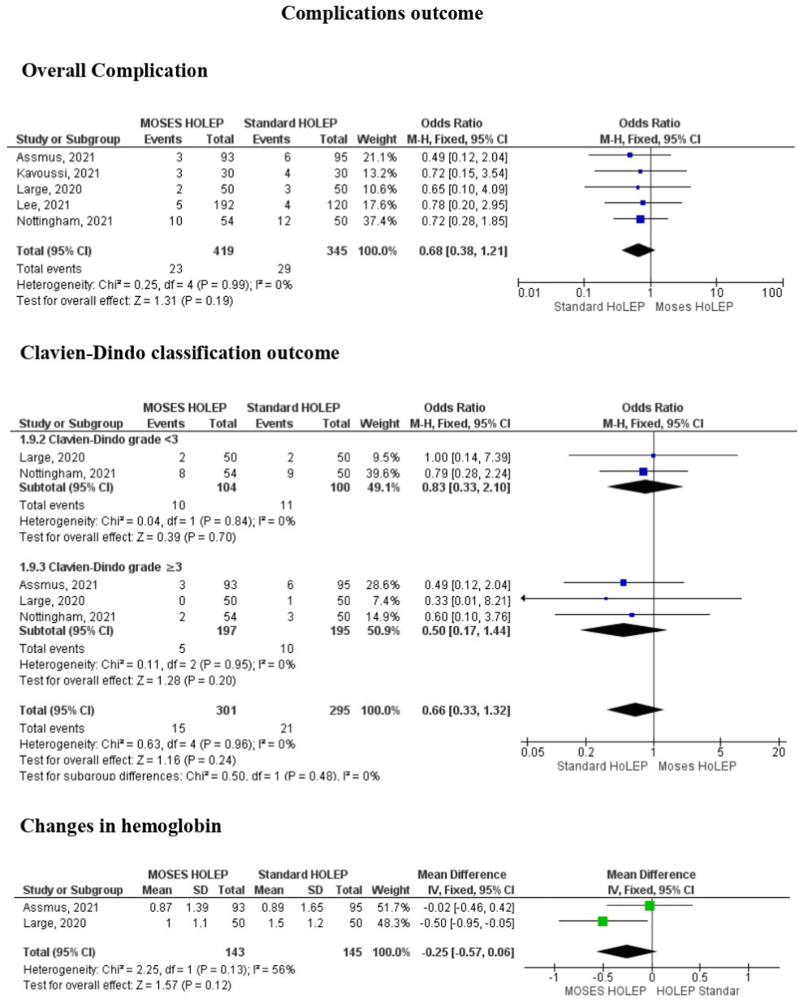
Forest plot of complications outcome consisted of overall complication, Clavien-Dindo classification outcome, changes in hemoglobin.

The results revealed that no significant differences were found between Moses HoLEP and standard HoLEP with regards to overall complication (MD 0.68, 95%CI: 0.38 to 1.21, p = 0.19). Moreover, if the complications were subgrouped into Clavien-Dindo classification, the result also did not find any significant differences in less than 3 (MD 0.83, 95% CI: 0.33 to 2.1, p = 0.7) and more than 3 (MD 0.5, 95% CI: 0.17 to 1.44, p = 0.2). However, there was likely a trend that Moses HoLEP possessed less complications compared to standard HoLEP. Hemoglobin change between Moses HoLEP and standard HoLEP also did not significantly differ between the two groups (MD: 0.25, 95% CI: 0.57 to 0.06, p = 0.12). Table 2 and Fig. 4 showed the detailed complications rate analysis.

**Table 2:** Complications rate between Moses HoLEP and standard HoLEP.

Study	Complications rate	
	Moses HoLEP	Standard HoLEP
Kavoussi NL (2021)	Cystitis (6.6%), urinary retention (3.3%)	Cystitis (6.6%), urinary retention (3.3%), syncope (%)
Nevo A (2020)	Deep vein thrombosis (1.7%), urinary tract infection (1.7%), epididymitis (1.7%), hematuria (1.7%)	
Large T (2020)	Clot urinary retention (1%), hematuria (2%), urinary tract infection (1%)	
Nottingham CU (2021)	Urinary retention (1.8%), urinary tract infection (14.8%), urethral stricture (1.8%), bladder neck contracture (3.7%)	Urinary tract infection (16%), clot urinary retention (2%), urethral strictures (2%)
Klett DE (2021)	NR	
Mark A. Assmus (2021)	Clavien-Dindo complications ≥ 3b = 3 (6%)	Clavien-Dindo complications ≥ 3b = 6 (12%)
Matthew S. Lee (2021)	Urinary retention (15%), hematuria (30%), UTI (5%), gastrointestinal (15%), respiratory (5%), musculoskeletal (5%), neurological (10%)

### 3.6 Meta-regression

**Fig. 5:**
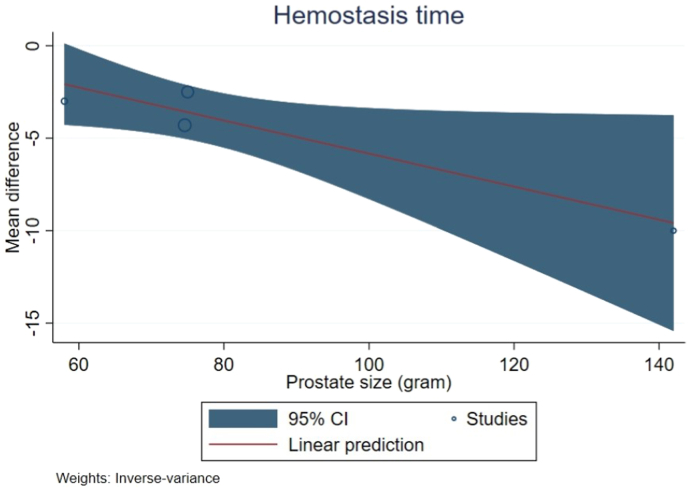
Meta regression of hemostasis time and prostate size.

The relationship between intraoperative procedure and prostate size was evaluated in meta-regression analysis. Our result revealed a significant association which Moses HoLEP possessed a more superiority over standard HoLEP regarding shorter hemostasis time with the increasing of prostate size (coefficient = −0.894, p = 0.044). The detailed analysis of meta-regression was shown in Table 3 and Fig. 5.

**Table 3:** Meta regression between intraoperative characteristic and prostate size (gram).

Outcome	Coefficient.	SE	95% Confidence Interval		P value
Enucleation time	−0.04497	0.0838	−0.2093	0.1193	0.592
Hemostasis time	−0.0894	0.044	−0.176	−0.0023	**0.044**^a^
Laser use time	−0.198	0.1376	−0.4686	0.0710	0.149

a Significant, SE: standard error.

### 3.7 Publication bias assessment

Publication bias was assessed using funnel plot, Egger regression test and Begg rank correlation test. The results of funnel plots were symmetrical in almost all intraoperative outcome except for total operative time (Supplementary materials). Egger regression test showed the result of 0.173 and Begg rank correlation test revealed the result of 0.188 in almost all studies. All these analyses revealed that there were no significant publication biases detected in this meta-analysis. However, in total operative time outcome, the asymmetry of funnel plot and inconsistency of sensitivity analysis suggested that this outcome may have significant publication bias.

## 4 Discussion

To the best of our knowledge, this is the first systematic review, meta-analysis and meta regression study to evaluate the comparison between Moses HoLEP laser technology and standard HoLEP in BPH patients. The HoLEP procedure is an effective treatment option for BPH specifically in large prostates [18,19]. Although holmium laser technology has existed for 25 years, the use of HoLEP technology was considered suboptimal in BPH patients. One of the reasons why this technology is still rarely used is because steep learning curve. The slow and longer learning process are complicated by the presence of bleeding, tissue damage, and scarring. Optimization of laser technology to overcome this problem may improve outcome and shorten the required time to master this technology [20].

The Moses HoLEP laser system forms the laser wave into 2 phases, the first phase was to separate the water by forming a bubble cavity and the second phase was to transfer the laser energy directly to the target [21]. This mechanism increased the amount of transferred laser energy to the target. This mechanism was also known as Moses effect. This mechanism was also explained from previous studies that the modulation of laser energy for enucleation process involves the formation of bubble cavity followed by the transfer of laser energy through the cavity and subsequently hit the targeted tissue [22,23]. The increased energy transfer by this technology increases the effectiveness of tissue ablation and hemostasis during the enucleation process, increases visibility, and makes it easier for surgeons to perform operations efficiently [6]. The results of this study suggested that Moses HoLEP significantly shorten the enucleation time, hemostasis time, and laser use time in intraoperative procedure. Moses HoLEP also showed superiority over standard HoLEP which Moses HoLEP possessed shorter hemostasis time with the increasing of prostate size. Moreover, in postoperative parameter, Moses HoLEP also possessed significantly less PVR compared to standard HoLEP.

In intraoperative outcome, our result suggested a superiority of Moses HoLEP in intraoperative outcome which Moses HoLEP possess the ability to shorten enucleation time, hemostasis time, and laser use time. The increased energy transfer via the 2-wave mechanism in this technology leads to an increase in the effectiveness of tissue ablation and hemostasis during the enucleation process, thereby increasing visibility and making it easier for surgeons to perform operations more efficiently. The shorter time in intraoperative procedure also represents the benefit of lowering the total cost required during this procedure [24]. The shorter time in Moses HoLEP is also associated with a lower incidence of capsular edema and a high degree of hemostasis, which may decrease the incidence of TWOC (Trial Without Catheter) failure which could lead to catheter reinsertion [15]. The increased energy transfer was believed to be the reason for increasing HoLEP efficiency and reducing total operative time and blood loss [21,25]. The presence of intraoperative bleeding is one of the factors that can prolong the duration of surgery. The importance of the ability to achieve hemostasis is required to speed up the intraoperative duration. This Moses technology has better hemostasis capability allowing a lesser energy used by the surgical operator. Moreover, longer intraoperative duration on the use of Moses HoLEP could also be due to the factor of surgical operator skill. The interaction between the laser fiber and prostate tissue is crucial for achieving enucleation. The goals of HoLEP are to rapidly identify the prostate capsule, maintain the surgical field, and maintain hemostasis during the procedure [26]. The acceleration of enucleation time in Moses HoLEP can also be explained that the enucleation efficiency in standard HoLEP was 1.05 g/min but increased to 1.75 g/min after using Moses technology [11]. This meta-analysis reported the result that Moses HoLEP had a faster time to hemostasis. Another study reported the use of Moses HoLEP accelerated hemostasis by 40% compared to standard HoLEP [7]. Another interesting result was also derived from our meta-regression analysis which Moses HoLEP showed an increasing advantage over standard HoLEP with the increasing of prostate size. This finding was also in accordance with multivariate analysis in a 1-year retrospective study from Large et al. which involved 150 patients. The study reported that for every 10g increase in prostate gland size, approximately 40% additional time is required to achieve hemostasis with standard 550μ HoLEP. However, this hemostasis time was 3.9 times faster when using laser technology with Moses laser modulation [7]. Moreover, laser use time was significantly shorter in the Moses HoLEP group than standard HoLEP. Shorter laser use time is associated with significantly faster enucleation and hemostasis times, thus reducing the duration of laser use. A non-significant result was observed in total operative outcome in this study possibly due to the influence of surgical operator in a 3 years of retrospective study from Klett et al. involving 487 patients [15]. They reported that the prolonged duration of total operative time in their study was significantly influenced by the surgical operator experience and skill in which the operator in their study performing Moses HoLEP procedure was a trainee. Moreover, we also did not include study from Assmus et al. in the analysis of total operative time because this study did not represent net operative time of Moses HoLEP procedure which the enucleation time and morcellation time comprised only 48.5% from the total operative time and many concurrent surgeries prolonged the total operative time [27]. Additionally, the total operative time analysis in this study possessed significant publication bias therefore the result was not conclusive.

The assessment of postoperative outcome comparison was also performed in this study. Lower mean postvoid residual volume was noted in Moses HoLEP group. This result could provide interesting evidence which Moses HoLEP group may represent favorable postoperative outcome compared to standard HoLEP. This is possibly due to the shorter intraoperative time and decreased intraoperative bleeding which may ease the surgeon to perform a better prostate enucleation with better visualization. The advantage of better intraoperative procedure may result in better postoperative outcome in BPH patients undergoing Moses HoLEP.

Moreover, the complication rates were also evaluated in this study. In terms of bleeding related outcome, Moses HoLEP did not significantly different compared to standard HoLEP. Hemologbin change was important parameter to be evaluated because this outcome was associated to bleeding related complications and may determine the need for blood transfusion. The overall complications are higher in standard HoLEP patient compared to Moses HoLEP eventhough the result was not significant. This result suggested that Moses HoLEP did not possess safety issues when compared to standard HoLEP. When sub-grouped to Clavien-Dindo classification, grade 3 complications requiring hospitalization were higher in standard HoLEP group although the result was not significant. The observed complications ranged from not requiring hospitalization such as urinary tract infection, cystitis to requiring hospitalization such as hematuria, urethral stricture, and bladder neck contracture.

The cost-analysis in this study was not able to be quantitatively assessed. However, one of included studies reported the cost comparison between Moses HoLEP and standard HoLEP [16]. They reported that the use of Moses HoLEP demonstrated significant hospital cost-savings of $840 per case of one initial surgical episode compared to standard HoLEP. In terms of visits and readmissions, Moses HoLEP also demonstrated hospital cost saving by $3220. Most of the Moses HoLEP patients who readmitted in ED were minor and did not require admission. Overall, Moses HoLEP represents a cost saving of $747 lower compared to standard HoLEP. The cost-saving of Moses HoLEP was possibly due to shorter intraoperative procedure times which leads to reduced medical and drugs supplies cost.

This study provided evidence on the enhanced efficacy and considerable safety of Moses HoLEP compared to standard HoLEP. Moses HoLEP represents a more effective operative procedure with promising outcome. This finding may shift future direction in the use of HoLEP into Moses HoLEP in the hope to optimize outcome in large BPH patients. This new technology will help surgeons to fulfill patient demand for important treatment, provide superior clinical outcomes, and elevate their practices. The practice of same-day discharge for BPH patients by using Moses HoLEP technology may also be fulfilled. The nature of lower cost in the use of Moses HoLEP may relieve the burden of the hospital in the service of large BPH treatment.

This systematic review is not without limitations. Firstly, only 2 RCT studies were included in this study and the other 5 studies were retrospective studies therefore the result of this study was still influenced under the nature of retrospective studies. Moreover, the level of evidence is higher for RCT studies compared to observational studies. Secondly, the influence of surgeon's skill and experience have not been able to be controlled in this study. Thirdly, the quantitative analysis of cost for the Moses HoLEP technology has not been able to be carried out in this systematic review due to limitation of primary thus future suggestion regarding cost analysis needs to be performed in future studies.

## 5 Conclusion

Moses HoLEP showed superiority compared to standard HoLEP. This study stresses the advantage of Moses HoLEP over standard HoLEP in intraoperative, postoperative and complications outcome. Moses HoLEP significantly provided shorter enucleation time, shorter hemostasis time and shorter laser use time. Moses HoLEP also possessed significantly lower PVR. There were no safety issues in Moses HoLEP compared with standard HoLEP.

## Ethics committee approval

A systematic review does not require an ethical approval. The protocol of this systematic review was registered in PROSPERO (CRD42021266151)

## Financial Support

None.

## Informed consent

This review does not need an informed consent statement.

## Author contribution statement

**Research conception and design:** Muhammad Zaniar Ramadhani, Yudhistira Pradnyan Kloping, Ilham Akbar Rahman. **Data acquisition:** Muhammad Zaniar Ramadhani, Yudhistira Pradnyan Kloping, Ilham Akbar Rahman, Niwanda Yogiswara **Statistical analysis:** Muhammad Zaniar Ramadhani, Ilham Akbar Rahman, Niwanda Yogiswara. **Data analysis and interpretation:** Muhammad Zaniar Ramadhani, Yudhistira Pradnyan Kloping, Ilham Akbar Rahman, Johan Renaldo, Soetojo **Drafting of the manuscript:** Muhammad Zaniar Ramadhani, Ilham Akbar Rahman. **Critical revision of the manuscript:** Muhammad Zaniar Ramadhani, Yudhistira Pradnyan Kloping, Ilham Akbar Rahman, Niwanda Yogiswara, Johan Renaldo, Soetojo. **Obtaining** funding**:** Not applicable. **Administrative, technical, or material** support**:** Muhammad Zaniar Ramadhani. **Supervision:** Johan Renaldo, Soetojo. **Approval of the final manuscript:** Muhammad Zaniar Ramadhani, Ilham Akbar Rahman, Yudhistira Pradnyan Kloping, Johan Renaldo, Soetojo.

## Provenance and peer review

Not commissioned, externally peer-reviewed.

## Consent

A systematic review does not require informed consent.

## Registration of research studies

Name of the registry: PROSPERO, Research Registry.

Unique Identifying number or registration ID: CRD42021266151 (PROSPERO), reviewregistry1403 (Research registry).

Hyperlink to your specific registration (must be publicly accessible and will be checked): PROSPERO: https://www.crd.york.ac.uk/prospero/display_record.php?RecordID=266151Resarch Registry:https://www.researchregistry.com/register-now#registryofsystematicreviewsmeta-analyses/registryofsystematicreviewsmeta-analysesdetails/62d5b21d704f1f001f4d4938/

## Guarantor

Johan Renaldo.

## Declaration of competing interest

All contributing authors have no conflicts of interest to declare. The signed ICMJE forms of each author were uploaded to the online system.
