# Supplementary material for: Comparative efficacy and safety of holmium laser enucleation of the prostate (HoLEP) using moses technology and standard HoLEP: A systematic review, meta-analysis, and meta-regression
Source: Ann Med Surg (Lond). 2022 Aug 12;81:104280. doi: 10.1016/j.amsu.2022.104280 (PMC9486436; doi:10.1016/j.amsu.2022.104280)
Supplement: Multimedia component 2 [file mmc2.docx]

**Supplementary materials**

**Figure 1**. Funnel plot of intraoperative outcome


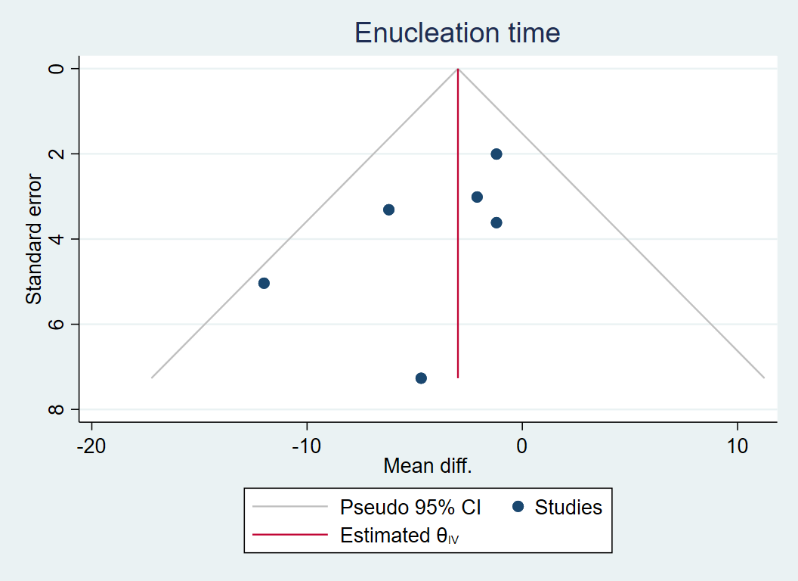

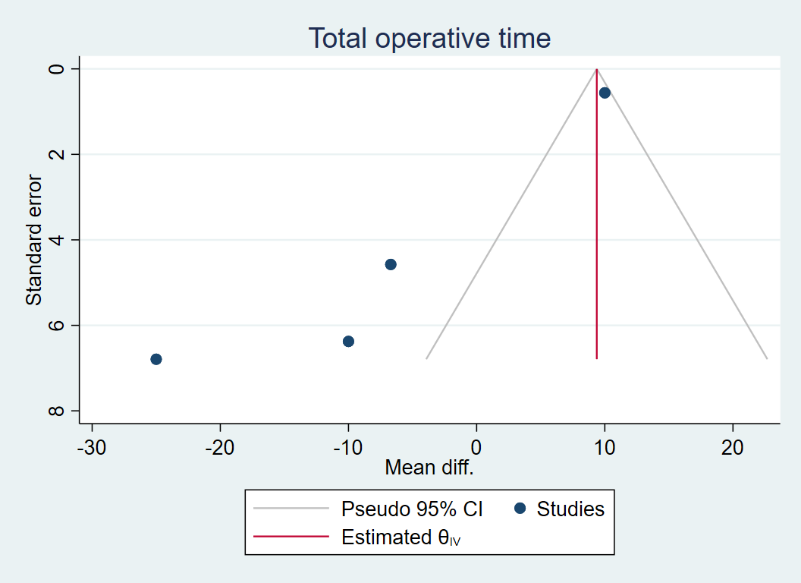

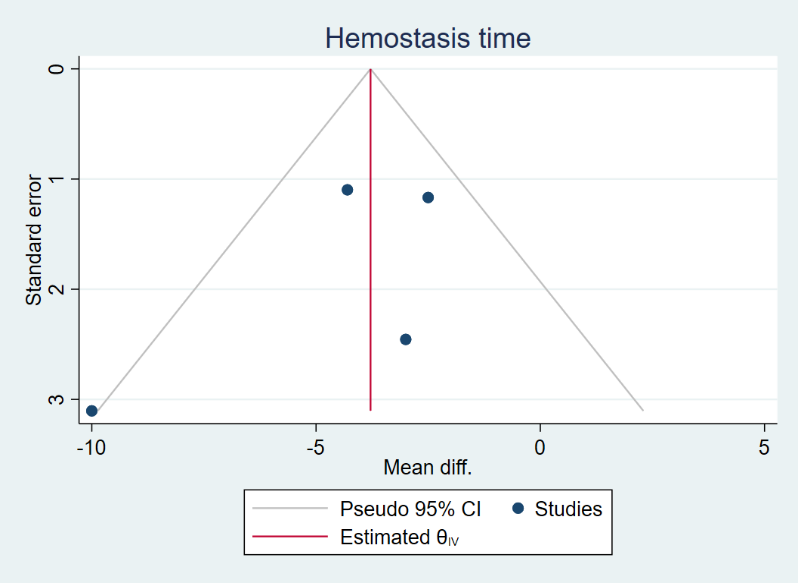

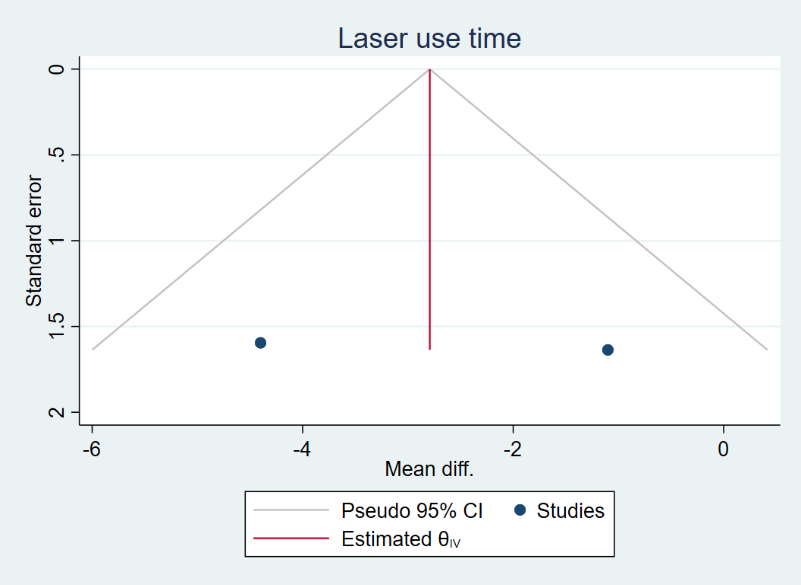


**Table 1**. Risk of Bias assessment of Included studies


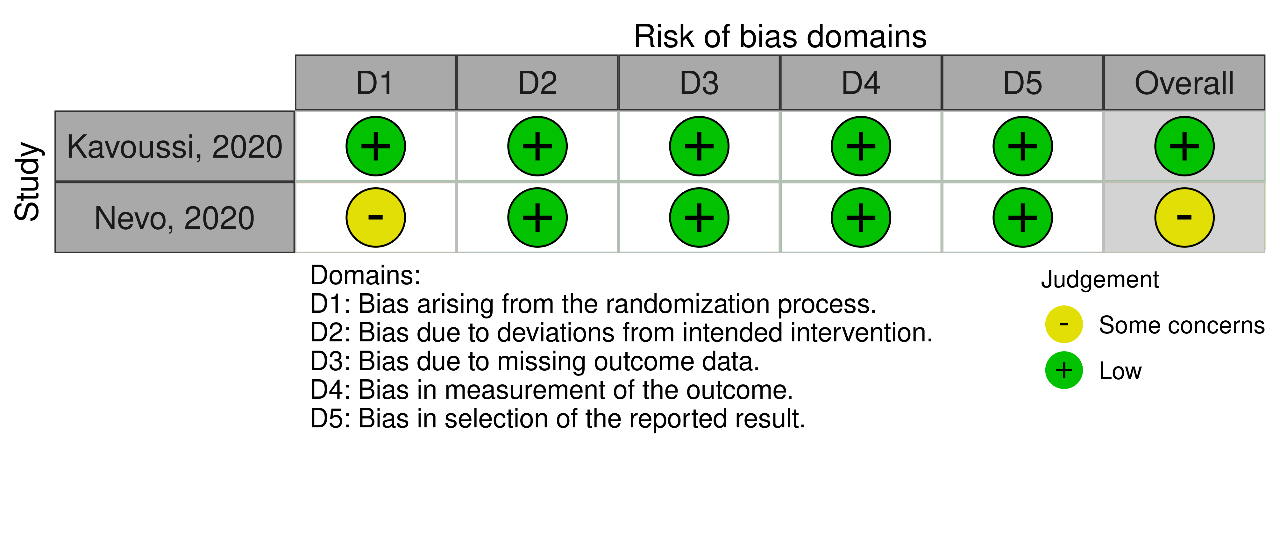


| **No** | **Authors** | **Years** | **Study Design** | **Quality score** | | | |
| --- | --- | --- | --- | --- | --- | --- | --- |
|  |  |  |  | **Selection** | **Comparability** | **Outcome** | **Total** |
| 1 | Nottingham, *et al* | 2021 | Retrospective study | 3 | 1 | 2 | 6 |
| 2 | Large, *et al* | 2020 | Retrospective study | 3 | 1 | 3 | 7 |
| 3 | Assmus, *et al* | 2021 | Retrospective study | 3 | 0 | 3 | 6 |
| 4 | Klett, *et al* | 2014 | Retrospective study | 3 | 0 | 3 | 6 |
| 5 | Lee, *et al* | 2021 | Retrospective study | 3 | 0 | 3 | 6 |

**Table 2.** Sensitivity meta-analysis summary for random effects model outcome (total operative time)

| Tau2: 0.25, I2: 2.11%, H2: 1.02 | | | | | | |
| --- | --- | --- | --- | --- | --- | --- |
|  | Mean Diff. | SE | z | P>\|z\| | [95% Conf. Interval] | |
| theta | -3.0277 | 1.33227 | -2.27 | 0.023 | -5.6389 | -0.4165 |
| Tau2: 104.4367, I2: 90%, H2: 10.00 | | | | | | |
|  | Mean Diff. | SE | z | P>\|z\| | [95% Conf. Interval] | |
| theta | -4.3808 | 4.505229 | -0.97 | 0.331 | -13.2109 | 4.4492 |
